# Identification, evolution, and expression analysis of *OsBSK* gene family in *Oryza sativa Japonica*

**DOI:** 10.1186/s12870-022-03905-1

**Published:** 2022-12-05

**Authors:** Shuo Zhang, Xuewei Hu, Jiejing Dong, Mengxiang Du, Juqi Song, Shangyuan Xu, Changjiang Zhao

**Affiliations:** 1 College of Agriculture, Heilongjiang Bayi Agricultural University, Daqing, 163319 Heilongjiang China; 2 Engineering Research Center of Crop Straw Utilization, Heilongjiang Province, Daqing, 163319 Heilongjiang China; 3 Key Laboratory of Low-carbon Green Agriculture in Northeastern China, Ministry of Agriculture and Rural Affairs, Daqing, 163319 Heilongjiang China; 4 Heilongjiang Provincial Key Laboratory of Modern Agricultural Cultivation and Crop Germplasm Improvement, Daqing, 163319 Heilongjiang China

**Keywords:** *Oryza sativa* L., OsBSKs, Gene family, qRT-PCR, Evolution

## Abstract

**Background:**

As an essential component of the BR (brassinosteroid) signaling pathway, *BSK* (BR-signalling kinases) plays a vital role in plant growth, development, and stress regulation. There have been sporadic reports on the functions of members of this family in monocotyledonous model plant rice, but few reports have been reported on the phylogenetic analysis and gene expression profiling of the family genes.

**Results:**

In this study, a total of 6 *OsBSK* members were identified at the genomic level by bioinformatics methods, distributed on four rice chromosomes. Through the evolution analysis of 74 BSK proteins from 22 species, it was found that BSKs originated from higher plants, were highly conserved, and could be divided into six subgroups. Among them, OsBSKs belonged to four subgroups or two significant groups. *OsBSK* family gene promoters contained a large number of light, abscisic acid (ABA), and methyl jasmonate (MeJA) response-related elements. At the same time, the qRT-PCR test also showed that the genes of this family were involved in response to a variety of hormones, biotic and abiotic stress treatments, and expression patterns of the family gene can be roughly divided into two categories, which were similar to the tissue expression patterns of genes in different growth stages. *OsBSK1–1*, *OsBSK1–2,* and *OsBSK3* were mostly up-regulated. *OsBSK2*, *OsBSK4,* and *OsBSK5* were mostly down-regulated or had little change in expression.

**Conclusions:**

This study revealed the origin and evolution of the *BSK* family and the farm-out of *BSKs* in rice growth, development, and stress response. It provides the theoretical reference for in-depth analysis of BR hormone, signal transduction, and molecular breeding design for resistance.

## Background

As the sixth class of hormones, brassinosteroid (BR) is a steroid hormone regulating plant growth and stress response [1, 2]. BR is widely present in various tissues and organs of plants, with varying contents of distribution in pollen, roots, stems, and leaves, indicating that BR can play an essential role in plant growth and development, such as seed germination, fibrous root formation, leaf morphogenesis, and flower organ differentiation [3]. Nawaz et al. point out that exogenous spraying of BR can not only enhance the drought resistance and salt resistance of plants but also reduce the incidence of rice blast, phytophthora root rot of soybean, and *Fusarium verticillioides* [4]. At the same time, it has also been fully proved at the molecular genetic level that the BR plays a vital role in regulating plant growth and development and stress. For example, the receptor BRI1 (Brassinosteroid insensitive 1) in the BR signaling pathway can regulate cell water uptake; the co-receptor BAK1 (BRI1 associated receptor kinase 1) has been proved to regulate plant height through expression level in *Oryza sativa* L.; BIN2 (BR-insensitive 2) can respond to cold stress; BZR1(Brassinazole resistant 1) can promote light response by regulating light signal-related genes [5–7]. In addition, some studies suggest that the BR signaling pathway may have emerged after the evolutionary divergence of vascular plants [8], and the components of the BR signaling pathway are remarkably conserved in the plant kingdom [9]. Therefore, the related research on plant BR hormone has essential theoretical and practical significance for producing different crops.

BSK(BR-signalling kinases) is a crucial component linking other components, which receives the signal from the BR receptor BRI1 and co-receptor BAK1 and then transmits the signal down to BIN2. BRI1 phosphorylates BSK and activates it. In this step, the leading site of BRI1 phosphorylates BSK is S230, and activated BSK then phosphorylates and activates BSU1 (BRI1 suppressor 1) [10, 11]. BSKs are plant-specific receptor-like cytoplasmic kinases belonging to a subfamily of the RLCK-XII superfamily, containing a TPR (Tetratricopeptide repeats) domain and a kinase PKc (Putative kinase catalytic) domain. Among them, the TPR domain has a binding function and can interact with other proteins [12]. The kinase PKc domain plays a vital role in plant cells’ differentiation, reproduction, and apoptosis. Studies have shown that Arabidopsis *bsk3* mutant reduced sensitivity to BR signaling, and other *bsk* single mutations have no significant effect on BR signaling; quadruple mutants *bsk3,4,7,8* and pentuple mutants *bsk3,4,6,7,8* all showed lower sensitivity to BR signaling [13]. The response of Arabidopsis BR to mild nitrogen deficiency in the formation of the root structure is mainly regulated by *BSK3* [14], and it is likely to regulate the signal in the form of a scaffold protein [15]. Arabidopsis BSKs can also, independent of BR signaling, interact with bacterial flagellin Flg22 (Flagellin peptide 22) and other multiple immune factors and participate in plant PTI (pattern-triggered immunity) responses [16–19]. In *O. sativa* L., studies have also found that *BSKs* not only can positively regulate rice BR signaling but also positively regulate rice immune resistance [20, 21]. *OsBSK2* can regulate granulotype independently of the BR signaling pathway [22]. The above studies show that *BSKs* have functional redundancy in the BR signaling transduction, and their family members may play different roles in different biological processes.

There have been many reports on the BR signaling pathway and the function of signal components [9, 23], but there are relatively few studies on the function of *BSK* genes. In rice, studies on the evolution and diverse functions of the *OsBSK* gene family have not been reported. This study started with the monocotyledonous model plant rice, and 6 *BSK* family members were identified at the genome level. The evolution analysis of different species found that *BSKs* originated from higher plants and were highly conserved. Furthermore, qRT-PCR was used to clarify the expression profiles of *BSK* genes in response to hormones, biotic, and abiotic stress, revealing their functional diversity. This study provides a theoretical basis for the origin and evolution of *BSKs*, reveals the role of *BSKs* in rice growth and development and response to stress, and provides a reference for the in-depth analysis of the BR signaling transduction pathway and application in crop production.

## Results

### Identification of the *OsBSK* gene family

**Fig. 1:**
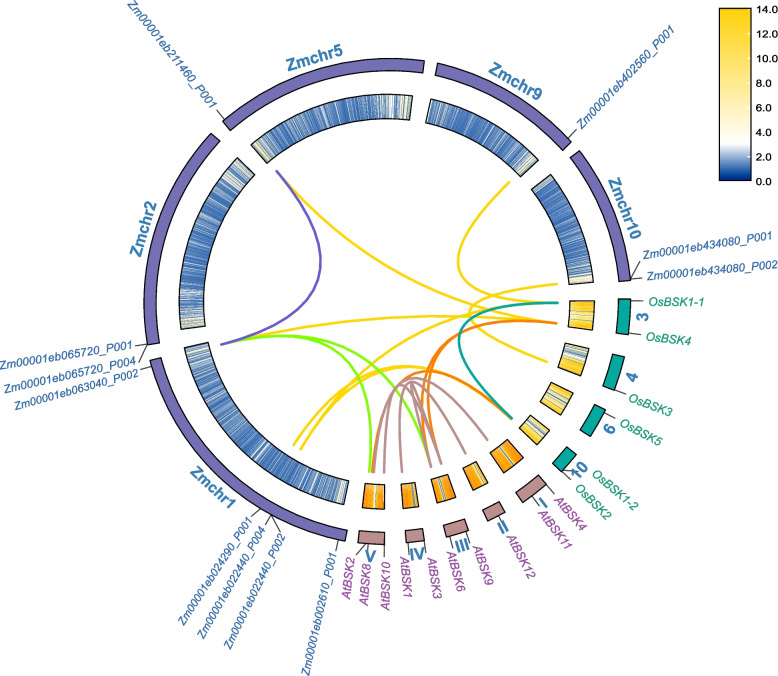
Chromosomal location and collinearity of *BSK* genes in *Oryza sativa Japonica*, *Arabidopsis thaliana*, and *Zea mays*. The blue, cyan, and purple lines indicate the collinearity of *Z. mays*, *O. sativa Japonica*, and *A. thaliana*, respectively. The yellow, orange, and green lines indicate the collinearity of *Z. mays* and *O. sativa Japonica*, *A. thaliana* and *O. sativa Japonica*, *Z. mays* and *A. thaliana*, respectively

A total of 6 *OsBSK* genes were identified at the rice genome level (Table 1), referred to previous literature named *OsBSK1–1* ~ *OsBSK5* [20], encoding amino acid chain lengths between 359 ~ 806 aa, molecular weights between 40.3 ~ 91.1 KDa, isoelectric points between 5.33 and 7.91. *OsBSK5* had the largest molecular weight and the lowest isoelectric point among them. As shown in Fig. 1, 6 *OsBSK* genes were located on chromosomes 3, 4, 6, and 10 of the 12 rice chromosomes, respectively. *OsBSK1–1* and *OsBSK4* were located on chromosome 3 together, and *OsBSK1–2* and *OsBSK2* were located on chromosome 10, relatively close. Subcellular localization prediction showed that most *OsBSKs* had nuclear and cytoplasmic localization.

**Table 1:** Basic characteristics of the *OsBSK* genes

Gene Name	Rice Genome Gene Number	Coding Regions/bp	Amino Acid/aa	Molecular Formula	Molecular Weight/kDa	Isoelectric Point	Subcellular Localization Prediction
*OsBSK1–1*	LOC_Os03g04050	1596	532	C_2604_H_4075_N_743_O_785_S_32_	59.4	6.05	Nuc/Mit
*OsBSK1–2*	LOC_Os10g39670	1569	523	C_2559_H_4033_N_731_O_761_S_30_	58.2	7.91	Nuc/Mit
*OsBSK2*	LOC_Os10g42110	1539	513	C_2492_H_3924_N_686_O_750_S_22_	56.2	5.94	Cyt/Nuc
*OsBSK3*	LOC_Os04g58750	1077	359	C_1766_H_2769_N_485_O_547_S_22_	40.3	5.45	Nuc/Cyt
*OsBSK4*	LOC_Os03g61010	1467	489	C_2398_H_3741_N_673_O_716_S_23_	54.2	6.20	Mit/Cyt
*OsBSK5*	LOC_Os06g06760	2418	806	C_3963_H_6357_N_1143_O_1245_S_3_	91.1	5.33	Nuc/Cyt

*Nuc* Nuclear, *Cyt* Cytoplasmic, *Mit* Mitochondrial

### Evolution of the *BSK* gene family in plant

**Fig. 2:**
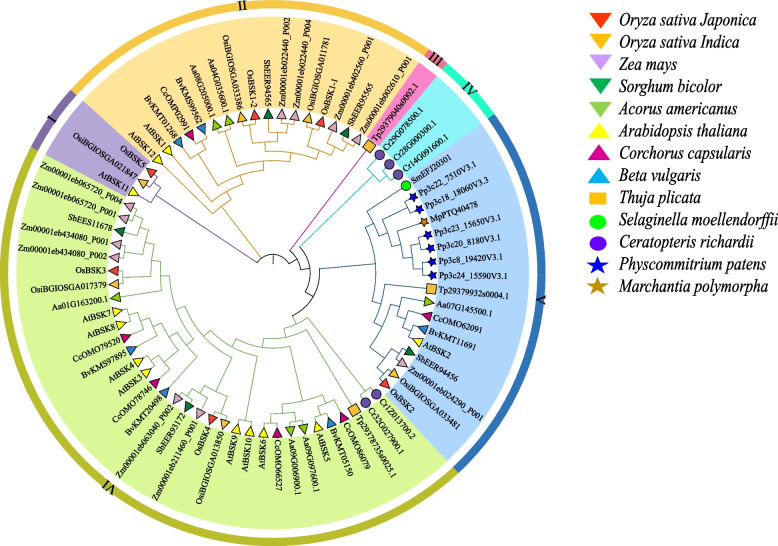
The phylogenetic tree of BSK proteins in plant. Consisting of a total of 74 BSK proteins from 13 species, divided into 6 subgroups

**Fig. 3:**
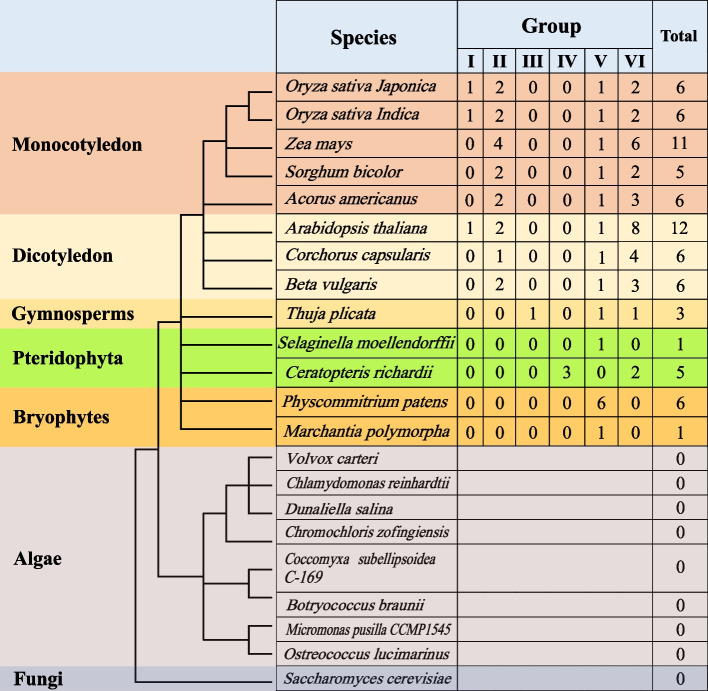
Species phylogenetic tree and number of *BSK* genes

As shown in Fig. 2, we isolated 13 species containing members of *BSKs* out of 22, all of which were higher plants. A total of 74 BSK proteins in these species were divided into six subgroups according to their evolutionary relationship. The V subgroup and the VI subgroup were located in the same evolutionary branch, and the evolutionary relationship was relatively close. Species of the same phylum were roughly equally distributed in different subgroups. Compared with other species, the BSK proteins in the two bryophytes *Physcommitrium patens* and *Marchantia polymorpha* were all located in subgroup V, indicating that this subgroup may be the early evolutionary form of the family. We identified six BSK members in *O. sativa Indica*. As shown in Fig. 2, these members could find member proteins with highly similar amino acid sequences in OsBSKs, but there was no member similar to the amino acid sequence of OsBSK5 except the homology in *O. sativa Indica*. The members of OsBSKs were divided into four subgroups. Subgroup I only had OsBSK5, subgroup II included OsBSK1–1 and OsBSK1–2, subgroup V only had OsBSK2, and subgroup VI included OsBSK3 and OsBSK4. Furthermore, combined with the evolution analysis of the rice BSK family, we could also divide them into two groups: OsBSK1–1, OsBSK1–2, and OsBSK5 were in the same group; OsBSK2, OsBSK3, and OsBSK4 were in the same group, of which OsBSK2 should be the origin of the rice BSK family. According to the structure and identification results of the phylogenetic tree, we counted the number of BSK members for each species, as shown in Fig. 3.

The results showed that *BSK* family members existed in bryophytes, pteridophytes, gymnosperms, and angiosperms and were more abundant in angiosperms, with more than five members in each species. However, these genes were still subjected to purifying selection and were evolutionarily conserved. In this study, no family member was found in algae and fungi, and only the PKc and TPR domain existed independently. Therefore, we speculated that *BSK* genes originated from higher plants, and the two specific conserved domains evolved gradually from a single PKc or TPR domain with the evolution of the plant.

In addition, the conserved domains of BSKs in various species except PKc and TPR were analyzed (Table 2), and almost all of the tested species contained PLN00113, which belongs to the leucine-rich repeat receptor-like kinase. STYKc was a phosphotransferase that occurs in conserved domains of three species. PLN03088 (SGT1) appeared in the conserved domains of *Thuja plicata* and *Beta vulgaris*, is a suppressor of the G2 allele of *skp1*, and could interact with RAR1 and HSP90 to participate in plant disease resistance responses [24].

**Table 2:** Conserved domains of various species

Classes	Species	Auxiliary Domains^**a**^
Bryophytes	*Physcommitrium patens*	STYKc
Pteridophyta	*Ceratopteris richardii*	PLN00113
Gymnosperms	*Thuja plicata*	PLN00113, PLN03088
Monocotyledon	*Acorus americanus*	STYKc, PLN00113
	*Oryza sativa Indica*	STYKc, PLN00113
	*Sorghum bicolor*	PLN00113
	*Zea mays*	PLN00113
Dicotyledon	*Arabidopsis thaliana*	PLN00113
	*Beta vulgaris*	PLN00113, PLN03088
	*Corchorus capsularis*	STYKc, PLN00113

^a^ The domains are located outside of the Pkc domain and TPR domain

### Analysis of *OsBSK* gene structure and their encoded protein structure

**Fig. 4:**
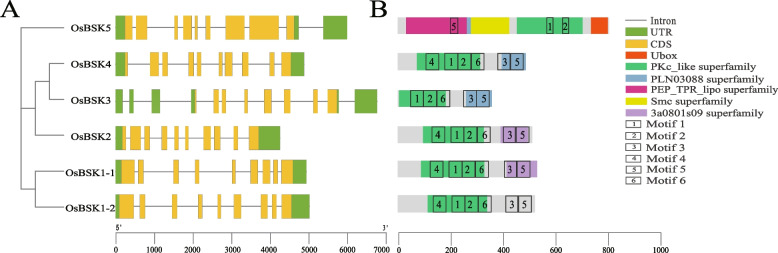
OsBSK gene structure, domains, and motifs in *Oryza sativa Japonica*. **A** *OsBSK* gene structure. **B** OsBSK domains and motifs. Motifs are arranged in the corresponding domains according to their position in the protein

Analysis of the intron and exon structure of the *OsBSK* family genes (Fig. 4-A) showed that the number of exons in each gene of the *OsBSK* family was 8–10. The *OsBSK2*, *OsBSK4*, and *OsBSK3* genes in the same group had different numbers of exons. The first two genes had ten exons, and the latter had eight exons; other *OsBSK* genes belonging to the same group had nine exons, and the *OsBSK1–1* and *OsBSK1–2* genes had similar structures.

As shown in Fig. 4-B, The OsBSK family all contained PKc (PF07714) and TPR (PF13414) domains, the PKc domain was located at the C-terminal of the protein, and the TPR domain was located at the N-terminal of the protein. However, the positions of PKc and TPR domains in OsBSK5 protein were opposite to other protein domains. Through protein conserved sequence analysis, six motifs were found in the OsBSK family, and motif1 was highly conserved. Combined with protein domain analysis, motif1 and motif2 were conserved motifs in the PKc domain, and motif5 belonged to conserved motifs in the TPR domain. The secondary structure of OsBSK family proteins showed that the extension chain and helical structure were intertwined, and they together constituted the N-terminus of OsBSK proteins, but its C-terminus only had a helical structure. OsBSK5 protein had the most significant proportion of α-helices. Studies had shown that a TPR motif contained two antiparallel α-helices [25], so it was speculated that OsBSK5 had more α-helix structures because it contained more TPR motifs. OsBSK3 protein had the least number of motifs, which had also been confirmed in the tertiary structure of OsBSK proteins.

### *Cis*-elements analysis of *OsBSK* gene promoters

**Fig. 5:**
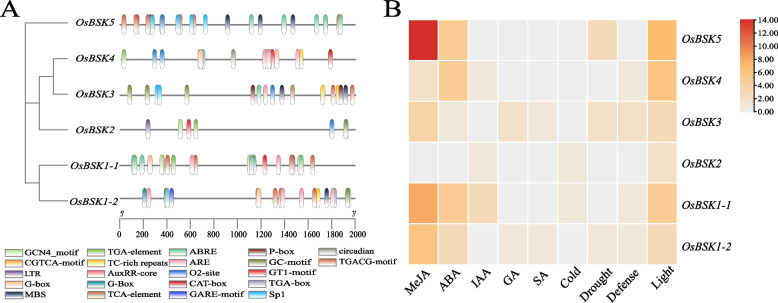
*OsBSK* elements and number statistics heatmap **A** The distribution of elements in *OsBSK*. **B** Heatmap of the number of *OsBSK* elements classified by role. Different colors represent different numbers of cis-elements

As shown in Fig. 5, the gene promoter sequence contained many light-responsive related elements, such as TGA-element, G-BOX, GT1-motif, and Sp1. Among the hormones, the number of acting elements of methyl jasmonate (MeJA) and abscisic acid (ABA) was more, and the acting elements of salicylic acid (SA), gibberellin (GA), and auxin were less. Anaerobic-related *cis*-elements ARE, drought-related *cis*-elements MBS, and low temperature-related *cis*-elements LTR were also found in abiotic stress. The number of growth and development-related elements was relatively small, such as O_2_-site, CAT-box, GCN4_motif, and circadian. It indicated that the *OsBSK* genes were involved in light response and the regulation of growth metabolism and were regulated by various hormones. However, it was worth noting that the types and numbers of tested *cis*-elements on the *OsBSK2* gene promoter were significantly less than those of other family members, and only this gene promoter had no *cis*-elements related to ABA response, which provided support for our previous speculation that *OsBSK2* was the origin of the *OsBSK* family.

### Analysis of proteins interaction with BSK

**Fig. 6:**
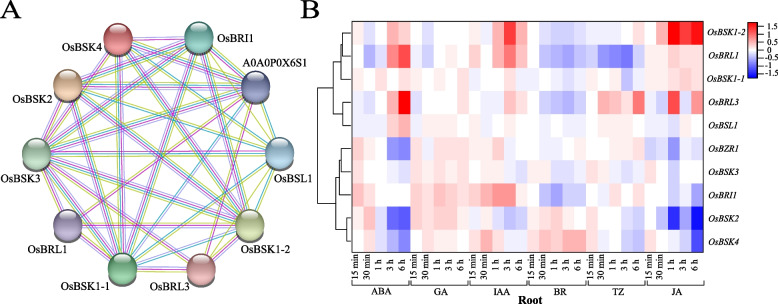
Protein interaction network prediction and gene expression heatmap. **A** Lines represent interaction relationships, where cyan, pink, gray, and green lines represent from curated databases, experimentally determined, protein homology, and textmining. **B** Gene expression heatmap with predicted protein interactions in rice seedling roots treated with ABA, GA, IAA, BR, trans-zeatin(TZ), and JA. Different colors represent different expression values

String prediction (Fig. 6-A) showed protein interaction among the five OsBSK (except OsBSK5). There were ten nodes and 35 groups of interaction relationships in the relationship network, among which OsBSK3 interacted with nine other proteins. In the interaction network, the five OsBSK members could interact with receptor kinases such as BRI1, BRL1, and BRL3; they could also interact with BSU1 family member BSL1. It was worth mentioning that A0A0P0X6S1 is the Os07g0503400 protein in japonica rice varieties, and the function of this protein was unknown. Yuan et al. have confirmed that there is a protein interaction between OsBSK2 and OsBSK3 in regulating grain size in rice, confirming our prediction [26]. As shown in Fig. 6-B, the expression of genes predicted to have protein interactions in the roots of rice seedlings treated with different hormones also proved the possibility of their interactions. For example, *OsBSK1–2*, *OsBRL1*, and *OsBRI1* had the same expression pattern after BR hormone treatment, indicating that they may interact in responding to BR hormone. The prediction results of phosphorylation sites showed that all family members had sites 39–71, and the number of threonines was significantly more than that of serine and tyrosine.

### Tissue expression profiles of *OsBSK* genes throughout the growth period

**Fig. 7:**
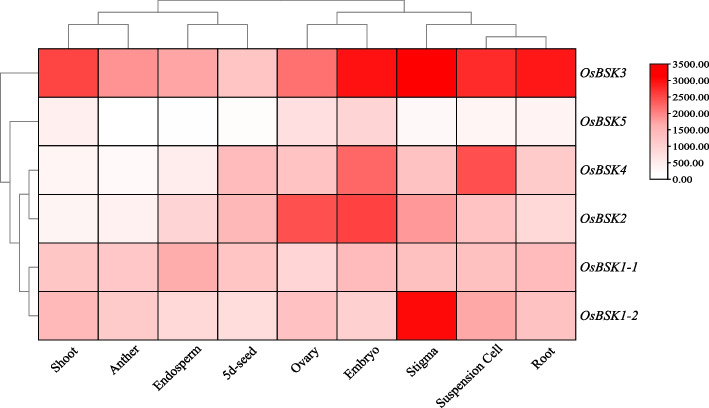
Expression profiles of *OsBSK* genes in the whole growth period. Different colors represent different expression values

The expression of *OsBSK* genes in different tissues during the whole growth period (Fig. 7) showed that *OsBSK* genes were expressed to different degrees in different developmental stages in *O. sativa* L.. Among them, *OsBSK3* was highly expressed during the whole growth period, and *OsBSK5* was low expressed during the whole growth period. The expression of *OsBSK1–1* and *OsBSK1–2* was similar, and the expression of *OsBSK2* and *OsBSK4* was similar. It could be seen that the expression of genes belonging to the same category in evolution was also roughly similar in the whole growth period.

### Expression analysis of *OsBSK* genes response to hormones

**Fig. 8:**
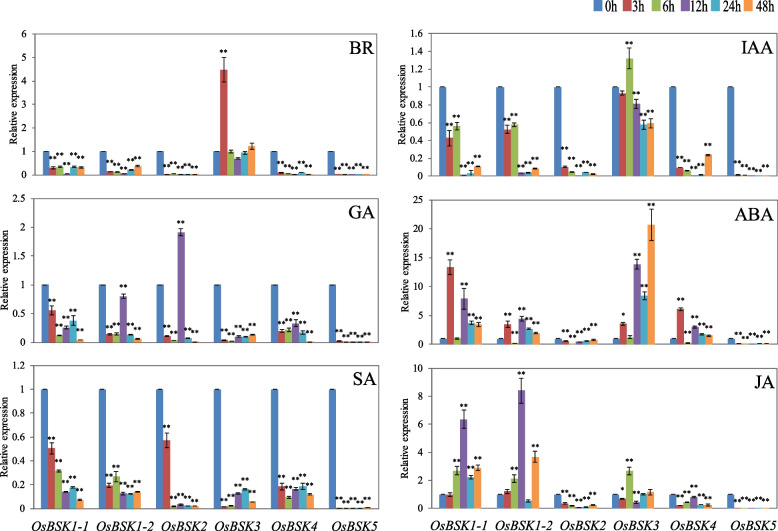
Expression analysis of *OsBSK* genes after hormones treatment with BR, IAA, GA, ABA, SA, and JA. (Significant differences: *, *P* < 0.05; **, *P* < 0.01)

As shown in Fig. 8, the expression patterns of *OsBSK* family members in rice seedlings treated with the six hormones could be roughly divided into two categories. *OsBSK1–1*, *OsBSK1–2*, and *OsBSK3* were up-regulated by at least two hormones; *OsBSK2*, *OsBSK4*, and *OsBSK5* were up-regulated by one hormone at most. After SA hormone treatment, all *OsBSK* genes were down-regulated; after BR, IAA, and GA hormone treatment, only one *OsBSK* gene was up-regulated, and the rest were down-regulated; after ABA and JA hormones, more than half of the *OsBSK* members were up-regulated. Among the six family members, *OsBSK5* had no expression changes in the six hormone treatments; *OsBSK3* could be up-regulated by ABA, JA, IAA, and BR hormones; *OsBSK1–1* and *OsBSK1–2* could also be up-regulated by ABA and JA hormones.

### Expression analysis of *OsBSK* genes under biotic and abiotic stress

**Fig. 9:**
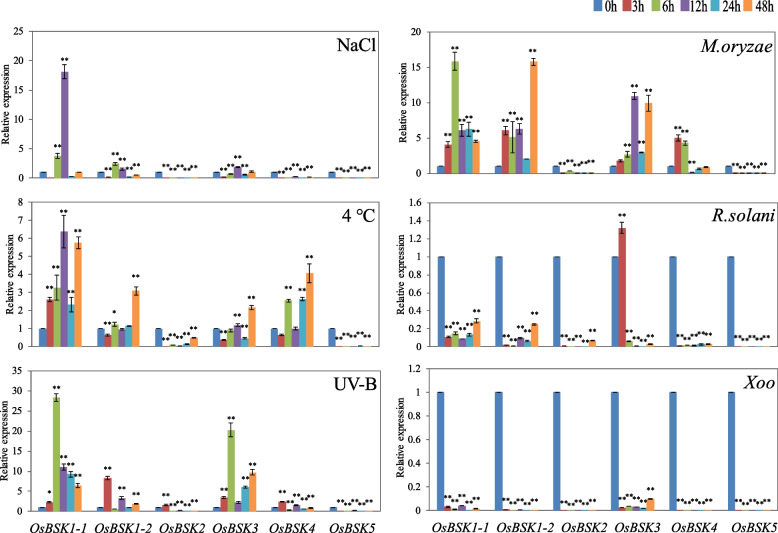
Expression profiles of *OsBSK* genes response to various stresses with NaCl, 4 °C, UV-B, *M. oryzae*, *R. solani*, and *Xoo*. (Significant differences: *, *P* < 0.05; **, *P* < 0.01)

As shown in Fig. 9, in the rice seedlings treated with abiotic stress such as NaCl, 4 °C, and UV-B radiation, the expression of *BSK* family members changed significantly, but the overall change trend was similar. *OsBSK1–1*, *OsBSK1–2*, and *OsBSK3* could be up-regulated by the three treatments tested. *OsBSK4* could be up-regulated by 4 °C and UV-B radiation and down-regulated by NaCl treatment. *OsBSK2* and *OsBSK5* showed no change or little change.

As shown in Fig. 9, the *OsBSK* family members differed greatly in response to inoculation of different pathogens such as *Magnaporthe oryzae*, *Rhizoctonia solani*, and *Xanthomonas oryzae pv oryzae*. In the rice seedlings inoculated with *M. oryzae*, the overall expressions of *OsBSK1–1*, *OsBSK1–2* and *OsBSK3* were up-regulated, while the expressions of *OsBSK2* and *OsBSK5* were down-regulated, and *OsBSK4* showed a noticeable change of up-regulation and then down-regulation. In the rice seedlings inoculated with *R. solani*, except for *OsBSK3*, which was up-regulated after 3 h of treatment, the other family member genes were down-regulated at different periods. In the rice seedlings inoculated with *Xoo*, the expression of all *BSK* family members was down-regulated at different periods.

## Discussion

### Identification and evolutionary analysis of the *OsBSK* family

In this study, six *OsBSK* gene family members were identified at the rice genome level, divided into four subgroups: subgroup I, subgroup II, subgroup V, and subgroup VI. Furthermore, we combined the evolutionarily related subgroups into two groups, namely OsBSK1–1, OsBSK1–2, and OsBSK5 as a group, and OsBSK2, OsBSK3, and OsBSK4 as a group. The multi-species phylogenetic analysis showed that *BSK* genes were generally more abundant in angiosperms, but no *BSK* genes were found in algae and fungi. It was speculated that this family of genes might originate from higher plants, which was mutually proved with the previous speculation that the BR signaling pathway appeared after the evolutionary divergence of vascular plants [8]. We found PKc and TPR single domains in algae and fungi and speculated that proteins containing only PKc or TPR domains in lower plants, coexisting PKc and TPR domains evolved in higher plants as plants evolved, the BSK family emerged and evolved in angiosperms, and underwent purification selection and were highly evolutionarily conserved. Even so, there are differences in *BSKs* in *O. sativa Japonica* and *O. sativa Indica*, for example, *OsBSK2 *[27].

In addition, according to the evolution analysis of different species, we speculated that subgroup V was the origin of family members in different species. OsBSK2 located in subgroup V may be the origin of OsBSKs. In our analysis of *cis*-elements in the promoters of *OsBSK* family genes, we found that only *OsBSK2* has no ABA-inducible elements, and the types and numbers of elements were the least, which can also provide support for our inferred view that *OsBSK2* was the origin of the family.

### Expression and function analysis of *OsBSK* genes

The protein interaction prediction analysis found that, except for *OsBSK5*, which was a low expression in the whole growth period, the other family members not only interacted with each other but also interacted with the upstream and downstream components of the BR signaling pathway, which was consistent with the OsBSK2 can directly interact with the BR receptor kinase OsBRI1 [22]. Indicating OsBSKs may play an essential role in the BR signaling pathway. Research has shown that Arabidopsis *bsk3* mutation could alleviate the inhibitory effect of exogenous BR on root growth, and *bsk5* mutation increased drought resistance and improved sensitivity to salt stress and ABA hormone [11, 28]. However, BSK3, 5, and 8 have functional redundancy. Arabidopsis mutant *bsk3, 4, 7, 8* and mutant *bsk3, 4, 6, 7, 8* can reduce the degree of leaf curl and increase leaf inclination [13]. We speculated that OsBSK3 or OsBSK4, which were classified in the same subgroup as the genes mentioned above, may have the same function. Previous studies has shown that *OsBSK1–2* is a significant positive regulator of rice plant immunity [20]. Because the gene structure and protein structure of OsBSK1–1 and OsBSK1–2 were highly similar, we speculated that these two genes might have similar functions. Meanwhile, *OsBSK1–1* and *OsBSK1–2* were up-regulated under salt stress and belonged to the same subgroup evolutionarily as *ZmBSK1* [29, 30]. We speculated that *OsBSK1–1* and *OsBSK1–2* also played an active role in the drought tolerance of rice plants. *OsBSK2* was expressed explicitly in ovary and embryo growth stages and was in the same subgroup as *AtBSK2* with known functions. We speculated that it was involved in the development of plant zygote and germ and stress responses of salt and high-temperature [31–33]. Recent studies have shown that this gene is involved in rice grain size [22], which also confirmed our inference.

In the analysis of *cis*-elements in the promoters of rice *BSK* genes, we found many light-responsive elements, MeJA-inducible elements, and ABA-inducible elements, indicating that hormones and light can regulate genes of this family. The expression analysis of *BSK* family genes in rice seedlings treated with hormonal, biotic, and abiotic stresses by qRT-PCR showed that the gene expression was in good agreement with the promoter prediction, especially the members of subgroups II and VI. In addition, the expression of *OsBSK2* in rice treated with ABA did not change significantly, which may be related to the absence of ABA-inducible elements in the promoter sequence of this gene.

Furthermore, we classified the expression patterns of 6 *OsBSK* genes and found that those treated with the hormone, biotic, and abiotic stress were the same and could be roughly divided into two groups: the majority induced up-regulated expression group and the expression down-regulated or no expression group. The former included *OsBSK1–1*, *OsBSK1–2*, and *OsBSK3*, which can be up-regulated by ABA, JA, UV-B, low temperature, and *M. oryzae*; The latter included *OsBSK2*, *OsBSK4*, and *OsBSK5*, most of which were down-regulated or unchanged in expression analysis except *OsBSK4* which could be up-regulated by ABA, low temperature and *M. oryzae*. Meanwhile, in the analysis of tissue expression profile during the whole growth period of rice, we found that *OsBSK1–1* and *OsBSK1–2* belonged to the same branch, and *OsBSK2* and *OsBSK4* belonged to the same branch, except for *OsBSK3* with high expression and *OsBSK5* with low or no expression. The abovementioned patterns of hormones and stress were generally consistent with the tissue expression patterns during the growth period. *OsBSK1–1* and *OsBSK1–2*, both of which belonged to the second subgroup, had the same expression pattern during the whole growth period, hormone, and stress treatment, making us more convinced of the conservation of family gene evolution.

In the expression profile of the tested hormones, ABA treatment induced up-regulation of 4 *OsBSK* genes, JA treatment induced up-regulation of 3 *OsBSK* genes, BR and IAA only up-regulated the *OsBSK3* gene, and SA treatment down-regulated the expression of all family genes. The *OsBSK3* gene was the most sensitive to exogenous BR and was up-regulated, consistent with previous results [21]. The above results indicated that *BSK* family members had both divisions and cooperation in response to different hormones and were involved in regulating plant growth, development, and stress. On the one hand, the responses of *BSKs* to the two hormones SA and JA were very different, which not only indicated that this family of genes may be involved in the defense response of plants to pathogens but also suggested that there may be different responses to different types of pathogens, such as biotrophic and necrotrophic pathogens. Our study also supported the inference that this family member induced distinctly different expression patterns upon infection by *M. oryzae*, *R. solani*, and *Xoo.* On the other hand, the *BSK* family was identified as a BR family, which had a more comprehensive response to the ABA hormone. Our study also supported this result. That is, different abiotic stresses in the test can cause the expression of family members to different degrees. Some *BSK* family genes in Arabidopsis can also be significantly changed by ABA, salt, cold, and high-temperature treatments [33]. Recently, studies at the genetic level have also shown that *ZmBSK1* plays an essential regulatory role in plant responses to drought and salt stress [29, 30].

## Conclusions

At the rice genome level, six members of the *OsBSK* family have been identified, which originated from higher plants and were highly conserved. These six genes can be divided into four subgroups or two major groups according to the conserved protein domain. The hormone and stress expression profiles of *OsBSK* family genes from the qRT-PCR assay had a certain relationship with their promoter *cis*-elements, and they also had a good similarity with the gene expression patterns during the growth period. The expression patterns of *BSK* family members in rice seedlings treated with six hormones (ABA, JA, SA, BR, IAA, and GA), three abiotic stress (NaCl, 4 °C, and UV-B radiation), and three pathogens (*M. oryzae*, *R. solani*, and *Xoo*) treatments were similar. Most of *OsBSK1–1*, *OsBSK1–2*, and *OsBSK3* were up-regulated; most of *OsBSK2*, *OsBSK4*, and *OsBSK5* were down-regulated or changed little.

## Methods

### Materials and treatment

Hormone and stress expression profiling analysis took the *O. sativa Japonica* variety “Kongyu 131” as the test material. Subsequent treatment was carried out when the rice in the culture medium grew to three leaves and one heart. At 0 h, 3 h, 6 h, 12 h, 24 h, and 48 h after treatment, the rice leaves were quick-frozen in liquid nitrogen and stored at − 70 °C for later use. Rice seeds were sterilized with 10% NaClO for 30 min, rinsed with distilled water three times, and germinated in the dark at 26 °C for 2 d. The seeds with the same bud length were selected and placed in the EP tube with the bottom cut off on the floating plate. The IRRI formula nutrient solution was changed every 3 d [34]. In the artificial climate room, the light intensity was 1200 μmol‧m^− 2^‧s^− 1^, the light time was 12 h/d, and the day and night temperatures were 28 °C/25 °C, respectively. Different hormones were sprayed on leaves at the concentrations of 0.5 mol/L IAA, 0.2 mol/L GA, 0.1 mol/L ABA, 0.001 mol/L BR, 0.2 mol/L SA, and 0.1 mol/L JA, respectively. For abiotic stress treatment, the hydroponic seedlings were placed in a constant temperature incubator at 4 °C, in a 150 mM NaCl solution, and under an 80 W UV lamp for 1 h before normal cultivation to complete low temperature, salt, and UV treatments. Appropriate inoculation methods were used for pathogen treatment. *M. oryzae* (strain 1391) was inoculated by spraying method, *R. solani* (strain YN-7) was inoculated by embedding method, and *Xoo* (strain XOOJ18) was inoculated by leaf clipping method. Each treatment was repeated three times.

### Identification of gene family members

The protein sequence alignment was performed in the NCBI database (http://www.ncbi.nlm.nih.gov/) according to the published protein sequences of Arabidopsis BSK. Meanwhile, the typical PKc and TPR domains were searched in the Rice Genome Database (http://www.ricedata.cn/gene/index.htm). Aligned and identified in the NCBI, SMART (http://smart.embl-heidelberg.de/) and Pfam (http://xfam.org/) databases to remove redundant proteins [35, 36].

The gene accession number, coding sequence length, and amino acid number were from the Rice Genome Database, and the molecular formula, molecular weight, and isoelectric point were from Expasy (http://web.expasy.org/cgi-bin/protaparam/protparam). Subcellular localization analysis was performed using Expasy, and chromosomal localization, gene density, and collinearity of genes were analyzed and visualized by TBtools software [37].

### Phylogenetic analysis of proteins

According to the published Arabidopsis BSK protein sequences, other species downloaded from the EnsemblPlants (https://plants.ensembl.org/index.html) and Phytozome (https://phytozome-next.jgi.doe.gov/) [38] databases were compared and screened by TBtools software and the NCBI website. The BSK family members of 13 species, including bryophytes(*Physcommitrium patens*、*Marchantia polymorpha*), pteridophytes(*Selaginella moellendorffii* 、*Ceratopteris richardii*), gymnosperms (*Thuja plicata*), monocotyledon(*Oryza sativa Japonica*、*Acorus americanus、Oryza sativa Indica、Sorghum bicolor、Zea mays*), and dicotyledon(*Arabidopsis thaliana*、*Beta vulgaris*、*Corchorus capsularis*) obtained after the screening, were used for sequence alignment and protein phylogenetic tree construction in MEGA X software. Selected the maximum likelihood method, set bootstrap to 1000, and applied the best fit model JTT + G. The evolutionary tree was beautified using Itol (https://itol.embl.de/) [39]. MEGA X and DNASP v6 software for Ka and Ks analysis [40]. Information on conserved domains of BSK members in various species was obtained from the NCBI database.

### Gene structure and protein structure analysis

Gene structure analysis of the *OsBSK* family was performed using TBtools. The motif of the OsBSK family protein was analyzed by MEME (http://meme-suite.org/tools/meme), and the protein secondary structure was predicted by prabi (https://npsa-prabi.ibcp.fr/cgi-bin/npsa_automat.pl?page=npsa_sopma.html) [41], and the protein tertiary structure was predicted by SwissModel (http://swissmodel.expasy.org/) [42]. In addition, the promoter sequence 2000 bp upstream of the gene translation initiation site was extracted and submitted to Plant CARE (http://bioinformatics.psb.ugent.be/webtools/plantcare/html/) [43] for *cis*-element analysis and used TBtools to make a statistical map of the number of cis-elements.

### Protein interaction prediction

The protein sequences of OsBSK family members were used to predict the protein interaction relationship through the string (https://cn.string-db.org/) database [44]. The expression data and heatmap of genes with predicted protein interactions under different hormone treatments were from the RiceXPro (https://ricexpro.dna.affrc.go.jp/) database [45]. NetPhos-3.1(https://services.healthtech.dtu.dk/service.php?NetPhos-3.1) was used to analyze the phosphorylation sites of the OsBSK proteins [46].

### Analysis of tissue expression profiling throughout the growth period

The expression data of the tested genes at different developmental stages were obtained from the GEO database (GSE7951) on the NCBI website [47, 48], and expression profiles were analyzed by TBtools software.

### Expression profile construction

Total RNA was extracted using the TRIZOL reagent (Invitrogen, Waltham, MA, USA). Then, the cDNA was synthesized through Fermentas (# 1622) reverse transcription kit for reverse transcription. The primer sequences of *BSK* genes and internal reference gene *actin* are shown in Table 3, which were synthesized by Sanbo Yuanzhi Company. Each gene was amplified three times, and the gene expression was calculated using the 2^-∆∆CT^ method [49]. Excel was used for data processing, GraphPad was used for histogram analysis, and SPSS 25.0 was used for significant difference analysis.

**Table 3:** qRT-PCR specific primers

Gene Name	Forward Primer Sequence (5′-3′)	Reverse Primer Sequence (5′-3′)
*OsBSK1–1*	ACAACCACAACACCCTCTATCC	CCTCATCTGCTGAGTCCATTCC
*OsBSK1–2*	AGCACAGTGTGTGTATCCCG	AGCTGTGATGCCTCGTTCAA
*OsBSK2*	TGCCAACTATTCTTTCTCCCCT	TGCACTTGCTGAGTCCATTCT
*OsBSK3*	GCCATGCCCTTGACCTGATT	ATCGCACTAGTTCTGTCCCTTC
*OsBSK4*	TGGTGTTGATGGACTCTTGCTT	AGAGCAGACACCACCGATTT
*OsBSK5*	AGATTGGAGAAGGTGGGTTTGG	ACCTCTTGTTCGAACTGTGATT
*OsActin*	CATGCTATCCCTCGTCTCGACCT	GCACTTCATGATGGAGTTGTAT

## Data Availability

All gene accession numbers referred to herein are provided in the corresponding tables in the text and can be obtained in the Rice Genome Database (http://www.ricedata.cn/gene/index.htm). The expression data of protein interaction genes are obtained from the RiceXPro database (https://ricexpro.dna.affrc.go.jp/). The tissue expression data of *OsBSKs* are from the GEO database (GSE7951).

## Abbreviations

BR: Brassinosteroid
BSK: BR-signalling kinases
Os: *Oryza sativa Japonica*
